# Decoding biomass recalcitrance: Dispersion of ionic liquid in aqueous solution and efficient extraction of lignans with microwave magnetic field

**DOI:** 10.1371/journal.pone.0226901

**Published:** 2020-02-21

**Authors:** Yu Xia, Jingdu Li, Zhijun Zhang, Sha Luo, Shouxin Liu, Chunhui Ma, Wei Li

**Affiliations:** 1 College of Material Science and Engineering, Northeast Forestry University, Harbin, China; 2 Key Laboratory of Bio-based Material Science and Technology, Ministry of Education, Northeast Forestry University, Harbin, China; 3 Institute of Urban Agriculture, Chinese Academy of Agricultural Sciences, Chengdu, China; Universidade de Aveiro, PORTUGAL

## Abstract

Alkaline ionic liquid aqueous solutions were used to extract biphenyl cyclooctene lignans derivatives, and hydrolyze to the free-state biphenyl cyclooctene lignans simultaneously from *Schisandra chinensis* by microwave-assisted heating. The hydrogen bonds formatted between ionic liquid and water molecular attacks the amorphous region of cellulose. Selective heating by microwave produce the more polar regions, which results in swelling and fragmentation of raw materials near the hot spots. Therefore, ionic liquid-microwave-assisted extraction method of free-state biphenyl cyclooctene lignans was set up. The solid residue after treatment was characterized by infrared spectroscopy and scanning electron microscopy, which showed that cellulose, hemicellulose, and lignin were removed partially. The water content of ionic liquid solution affected its viscosity and diffusivity, and in turns the extraction efficiency of lignans. The IL solutions with different mole fractions of IL were detected by FTIR and Raman spectroscopy, the result shows that IL solutions with higher water contents (>0.6) won’t form clusters. The optimum hydrolysis conditions were 0.2 g of ionic liquid catalyst per 5.0 g of *S*. *chinensis* fruits, a microwave irradiation power of 600 W, and heating time of 12 min, which gave a yield of free-state biphenyl cyclooctene lignans of 4.12±0.37 mg g^−1^. Besides, a hydrolysis mechanism of ester-bond biphenyl cyclooctene lignans and decreasing "biomass recalcitrance effect" by ionic liquid microwave-assisted method was proposed.

## 1. Introduction

Ionic liquids (ILs) are composed of bulky organic cations and inorganic or organic anions and have some unique properties such as negligible vapor pressure, nonflammability, high thermal and chemical stability, tunable chemical structures and physical properties, and strong solubilization of organic and inorganic compounds [1–3]. The potential alkaline sites of ILs containing Lewis basic anions can exhibit catalytic activity [4]. Thus, IL catalyst is designed through selection of the potential alkaline sites of ILs, which has been used in Friedel–Crafts acylation [5], Heck [6], oxidative desulfurization [7], cycloaddition [8], and hydrolysis reactions [9,10].

Plant cell walls are composed of three major components, cellulose, hemicellulose, and lignin. Cellulose is the major structural skeleton of the plant cell wall [11]. The linearity of cellulose provides close associations that allow each glycan chain to form intra- and intermolecular hydrogen bonds, leading to long, tightly packed microfibrils (semicrystalline structures) [12]. Hemicelluloses are a group of polysaccharides that interact with cellulose and lignin [13]. Plant cells are glued together by the middle lamella, an area with high lignin content. Because of the presence of lignin, the crystallinity of the cellulose, and the covalent cross links between lignin and hemicelluloses in the plant cell wall, they form physical barriers to protect intracellular active substances [14]. In previous literature, hydrophilic ionic liquid had been used in biomass pretreatment to disrupt the *Haematococcus pluvialis* thick cell wall structures and remove cellulose components to improve the extractability of astaxanthin [15]. Pretreatment of biomass with acid or alkaline ILs is environmentally benign and may offer several advantages over homogeneous acid or alkaline pretreatment in terms of total processing time and efficiency.

*Schisandra chinensis* (Turcz.) Baill (*S*. *chinensis*) is an important traditional Chinese medicine and edible natural product that has been used for 2000 years in China [16]. It grows in the Hebei, Heilongjiang, Jilin, Liaoning, Inner Mongolia, and Shanxi provinces of China, and in Russia, Japan, and Korea [17]. *S*. *chinensis* has been reported to increase vital energy and promote the production of body fluid, invigorate the kidneys, and calm mood [16]. The biphenyl cyclooctene lignan (BCL) compounds that exist in *S*. *chinensis* fruit have two states. Free-state biphenyl cyclooctene lignans (FBCLs) such as Schizandrin (S), Schisantherin A (SA), Deoxyschizandrin (DS), γ-Schizandrin (GS), and Schisandrin C (SC) display antioxidant, anti-aging, liver- and heart-protecting, and central nervous system-inhibiting effects [18–20]. The other is ester-bond biphenyl cyclooctene lignans (EBCLs), which has lower pharmacological activities than FBCLs [21]. EBCLs was reported can be hydrolyzed by an alkaline catalyst without inducing other structural changes [22]. In this study, microwave-assisted heating (MAH) is used to hydrolyze EBCLs. Recently, MAH has been accepted as an effective technique compared to conventional heating methods in natural product extraction [23]. Moreover, MAH boasts the advantages of being green, increasing yield, saving energy, shortening reaction time, and avoiding damage of active components at high temperature [24].

Previously published of our team concerning the extraction of FBCL from *S*.*chinensis* by using neutral imidazolium-based ILs and microwave- and ultrasound-assisted extraction [19,22]. In our work, Brönsted base ILs, which has two functions: decrease *S*. *chinensis* fruit cell wall “biomass recalcitrance” to make the BCLs dissolved, and hydrolyze EBCLs was used. The potential catalytic site, concentration of selected IL, and microwave irradiation power and time are systematically optimized. The mechanism of the hydrolysis reaction is considered in detail.

## 2. Materials and methods

### 2.1 Materials and reagents

*S*. *chinensis* fruits were purchased from Sankeshu Medicinal Materials Market, Harbin, Heilongjiang Province, China, and authenticated by Prof. Shao-quan Nie from the Key Laboratory of Forest Plant Ecology, Ministry of Education, Northeast Forestry University, China. Voucher specimen was deposited in the herbarium of this Key Laboratory. Dried fruits were powdered into a homogeneous size and then sieved (20–40 mesh) prior to use.

Standard samples (≥98% purity) of S (110857–201513), SA (58546-56-8), DS (110764–201513), GS (110765–201311), and SC (61301-33-5) were purchased from the National Institute for the Control of Pharmaceutical and Biological Products (Beijing, China). The ILs (≥99% purity) [C4mim]OH and [C4mim]Ac were purchased from Chengjie (Shanghai, China). Acetonitrile and acetic acid of high-performance liquid chromatography (HPLC) grade were purchased from J&K Chemical Ltd. (Beijing, China). Deionized water was purified using a water purification system (Milli-Q, Millipore, MA, USA). The other solvents and chemicals used in this study were of analytical grade and purchased from Beijing Chemical Reagents Co. (Beijing, China). All the solutions prepared for HPLC were filtered through 0.45 μm membranes (Guangfu Chemical Reagents Co., Tianjin, China) before use.

### 2.2 Methods

#### 2.2.1 FTIR spectroscopic analysis of IL aqueous solutions

[C4mim]OH and [C4mim]Ac aqueous solutions with mole fractions of 0.2, 0.4, 0.6, 0.8, and 1.0 were prepared and then detected by FTIR spectroscopy (Nicolet iS10, Thermo, USA). The FTIR spectra of the IL-water mixtures were measured from 600 to 4000 cm^−1^ with 4-cm^−1^ resolution for an average of 32 times. Each spectral measurement was repeated 3 times, and all measurements were performed at room temperature.

#### 2.2.2 Raman spectroscopic analysis of IL aqueous solutions

Raman spectra of the IL aqueous solutions in Section 2.2.1 were then collected by a Renishaw inVia Raman microscope (Renishaw, London, UK) in quasi-backscattering geometry using 5 mW of the 632.8-nm line of an argon-ion laser as the excitation source focused to a line of 5 mm×100 μm. The scattered light was filtered with a holographic edge filter, dispersed by a Spex 0.55-m spectrometer, and then detected with a liquid nitrogen-cooled back-illuminated charge-coupled device detector array. Both the spectral resolution and accuracy of the Raman shift were estimated to be approximately 2 cm^−1^. Each spectrum was the average of three measurements, and all measurements were conducted at room temperature.

#### 2.2.3 HPLC quantitative analysis of FBCLs

Chromatographic isolation was conducted on an HiQ sil-C18 reversed-phase column (4.6 mm×250 mm, 5 μm, KYA Technologies, Tokyo, Japan) to analyze FBCLs. Acetonitrile/water/acetic acid (60:40:2, v/v/v) was used as the mobile phase with a flow rate of 1.0 mL min^−1^, injection volume of 5 μL, and column temperature of 25 °C. The absorbance at 220 nm was measured to detect FBCLs and the run time was 40 min. The retention times of S, SA, DS, GS, and SC were 5.6, 11.4, 22.9, 30.4, and 36.4 min, respectively. Corresponding calibration curves for S, SA, DS, GS, and SC were *Y*_S_ = 24572*X*+659.03 (*r* = 0.9999), *Y*_SA_ = 11165*X*+130.09 (*r* = 0.9998), *Y*_DS_ = 3129.4*X*+42.664 (*r* = 0.9998), *Y*_GS_ = 3559.9*X*+22.195 (*r* = 0.9999), and *Y*_SC_ = 2777.2*X*+29.375 (*r* = 0.9998), respectively. Good linearity was found for S, SA, DS, GS, and SC in the ranges of 0.011–0.40, 0.0003–0.42, 0.018–0.77, 0.012–0.37, and 0.011–0.039 mg mL^−1^, respectively.

#### 2.2.4 Hammett acidity detection of IL aqueous solution

The acidity of the ILs was evaluated from the determination of the Hammett acidity functions using UV−visible spectroscopy. This method consists of evaluating the protonation extent of uncharged indicator bases (named I) in a solution, in terms of the measurable ratio [I]/[IH^+^], 2,4-dinitroaniline (p*K*(I) = −4.53) was chosen as the indicator, and the Hammett function (*H*_0_) is defined as

$$H_{0}=\text{p}K\left(\text{I}\right)+\text{log}\left(\left[\text{I}\right]/\left[\text{I}{\text{H}}^{+}\right]\right)$$

Equal volume mixing of ionic liquids and 2,4-dinitroaniline of the same concentration was evaluated using UV−visible spectroscopy to measure the absorbance A1 of the solution, control group without ionic liquid was evaluated to measure the absorbance A2 of the solution, [I] = A1/A2, [IH^+^] = 1-[I], and their acidity *H*_0_ was calculated by Hammett acidity functions.

#### 2.2.5 Preparation of *S*. *chinensis* BCL extracts

Dried *S*. *chinensis* fruit (100 g) in ethanol/water (1000 mL, 80:20, v/v) was heated at 90 °C for 2 h and then filtered. The residue was refluxed in ethanol/water (1000 mL, 80:20, v/v) for 2 h and then filtered. The two liquid extracts were combined for further use.

#### 2.2.6 MAH hydrolysis of EBCLs

*S*. *chinensis* extract (50 mL) was treated by the IL-MAH method. The potential alkaline anion, concentration of selected IL, and microwave irradiation power and time were systematically studied. The process was repeated three times for each set of conditions and the average value was recorded.

#### 2.2.7 Comparison of the IL-MAH method with traditional methods

*S*. *chinensis* fruit (5 g) was mixed with IL aqueous solution (50 mL) and treated by MAH under the optimal conditions (acidic anion, IL concentration, microwave irradiation power and time as selected in single factor analysis) to extract BCLs and hydrolyze EBCLs simultaneously.

*S*. *chinensis* fruit (5 g) was mixed with IL aqueous solution (50 mL) and then heated reflux for 2 h to extract BCLs and hydrolyze EBCLs simultaneously using the optimal conditions (acidic anion, IL concentration as selected in single factor analysis).

*S*. *chinensis* fruit (5 g) was mixed with ethanol/water (50 mL, 10:90, v/v) and then treated by MAH using the optimal conditions (microwave irradiation power and time as selected in single factor analysis) to extract BCLs.

All the cooled extracts were filtered through a 0.45-μm membrane for subsequent HPLC analysis. Each test was conducted three times and the average value was recorded.

#### 2.2.8 SEM analysis of *S*. *chinensis* fruit

*S*. *chinensis* fruit and *S*. *chinensis* fruit residue obtained from Section 2.2.6 were observed by SEM (FEI QUANTA 200, Netherlands). The sample surfaces were sputter coated with a thin layer of gold using an SCD 005 sputter coater (BAL-TEC, Switzerland) to provide electrical conductivity.

## 3. Results and discussion

### 3.1 State of [C4mim]Ac in aqueous solution

**Fig 1:**
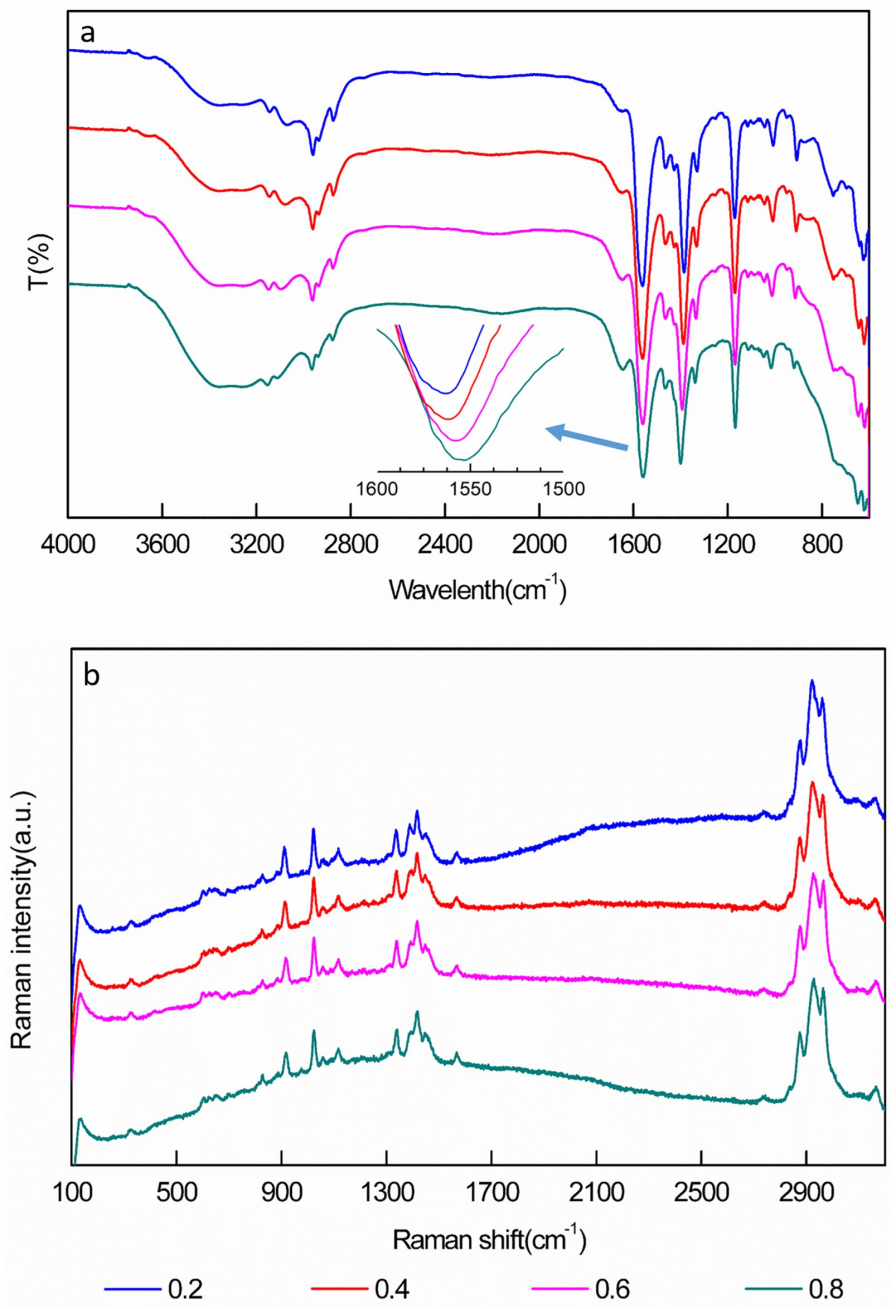
FTIR (a) and Raman spectra (b) of [C4mim]Ac in aqueous solutions with different IL/water mole fractions.

Water is a necessary component for extraction of BCLs from *S*. *chinensis* fruit. However, the mole fraction of water in IL solutions greatly affects the microstructure and dynamic properties of the IL, including self-aggregation behavior, density, surface tension, and especially viscosity, which strongly influences the diffusivity of IL aqueous solutions. The formation of clusters is a result of the combination of electrostatic and intermolecular forces, such as hydrogen bonding, π-bonding, polarization, but also non-electrostatic interactions [25]. FTIR spectroscopy is arguably the most powerful method to probe the molecular state of water present in various solvents. Different mole fractions of [C4mim]Ac in water were detected by FTIR spectroscopy. The spectra (Fig 1a) showed negligible changes in the imidazolium Ѵ(C-H) stretching region (3000–3200 cm^−1^) when the water mole fraction was 0.2 to 0.8. This indicates that there was no marked hydrogen bonding between H of the imidazolium ring protons and water, which implies that the anions of [C4mim]Ac play major roles in the solubility and miscibility of water in this IL, whereas the cations probably play a secondary role. Therefore, we concentrated on the OH stretching vibration region above 3200 cm^−1^ because it contained signals affected by structural changes of water and the IL anion. The broad band in the region around 3400 cm^−1^ originated from water interacting strongly with the COO^−^ group of the anion and water forming aggregates around this anion. The strong interaction between water molecules and the COO^−^ group of the anions was evidenced by the low frequency shift (10 cm^−1^) of the shoulder of the carboxyl band of this anion (Ѵ(COO^−^) band at 1550 cm^−1^) in the presence of water [26]. The association of water molecules and anions weakens the polar network of imidazolium rings and anions; that is, the C-H···O cation–anion interactions are replaced with hydrogen-bonding interactions between the anions and water.

Fig 1b shows the Raman spectra of [C4mim]Ac aqueous solutions with several different mole fractions of water to [C4mim]Ac. The polar heads of imidazolium-based ILs are known to form hydrogen-bonded ion pairs [27]. Hydrogen-bond interactions occur between the anions and C-H protons of the imidazolium rings, particularly the acidic proton C(2)-H in [C4mim]Ac. With increasing water content, the hydrogen-bonding interactions between ion pairs weakened and the number of hydrogen bonds between water molecules and anions increased. The stretching modes of the imidazolium-ring C-H groups, whose Raman scattering contributions appeared in the range of 3000–3200 cm^−1^, should be affected by this replacement. However, our measurements did not show appreciable changes in the positions or relative intensities for these bands with evolving water-IL mol fraction; this may be a consequence of the difficulty of spectral deconvolution because of the overlap between the C-H and O-H stretching regions. For mixtures with higher water contents, the Raman spectra display the rise of another scattering contribution centered at 3250 cm^−1^ that is attributed to the O-H stretching modes of water molecules that form four hydrogen bonds with other water molecules and are involved in a transient network. This band corresponds to the water molecules that behave as the bulk-like inner part of water aggregates. Water molecules form a hydrogen-bonding network with anions and displace the anions, thereby disturbing the stable arrangement of IL components [28].

**Fig 2:**
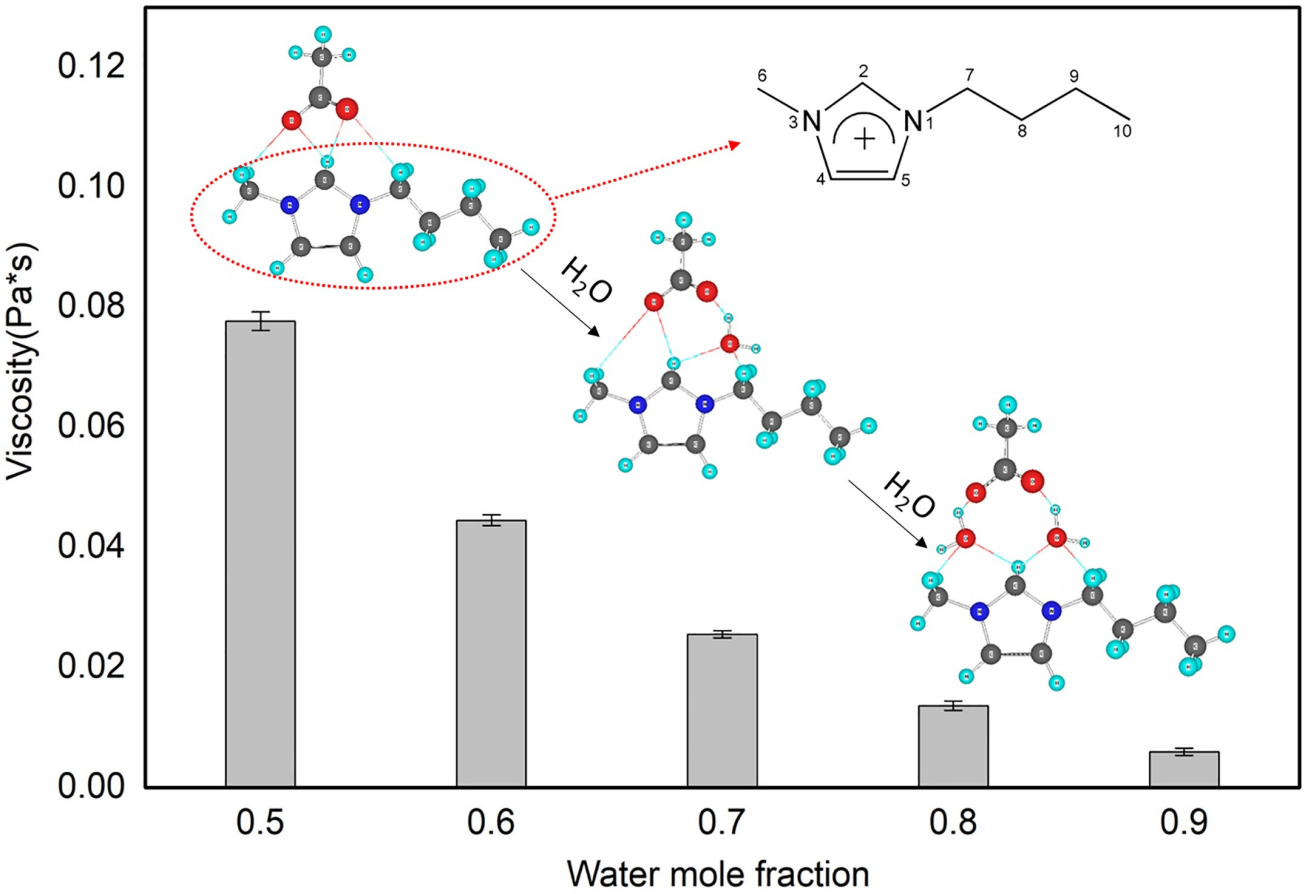
Viscosity and dispersion state of [C4mim]Ac aqueous solutions with different IL/water mole fractions.

According to the results of FTIR and Raman spectroscopic analyses, water molecules tend to interact with first the anions and then the cations of [C4mim]Ac [29]. Fig 2 illustrates how the extended hydrogen-bonding network between cations and anions of the pure IL is gradually degraded into ionic clusters and then the ionic clusters are further dissociated into solvent-surrounded ion pairs as the water content increases [30–32]. Finally, the ion pairs become the dominant form of [C4mim]Ac in bulk water. Meanwhile, small water clusters bound to both the anion and cation of the ion pairs interacted with each other, thereby forming a percolating network of "ice-like" water molecules in the mixtures [33,34]. From Fig 2, the viscosity of [C4mim]Ac decreased with increasing water content. A small amount of water had a considerable effect on the viscosity of [C4mim]Ac because hydrogen bonds formed between water molecules and the IL components. Water acted as a diluent in the system, which led to a decrease of viscosity and greatly increased the diffusivity of the IL aqueous solution. Therefore, the [C4mim]Ac solutions with high water contents should perform better in the extraction process than those with a lower water content.

### 3.2 Single factor analysis of hydrolysis experiments

#### 3.2.1 Screening of the basic IL solution

**Fig 3:**
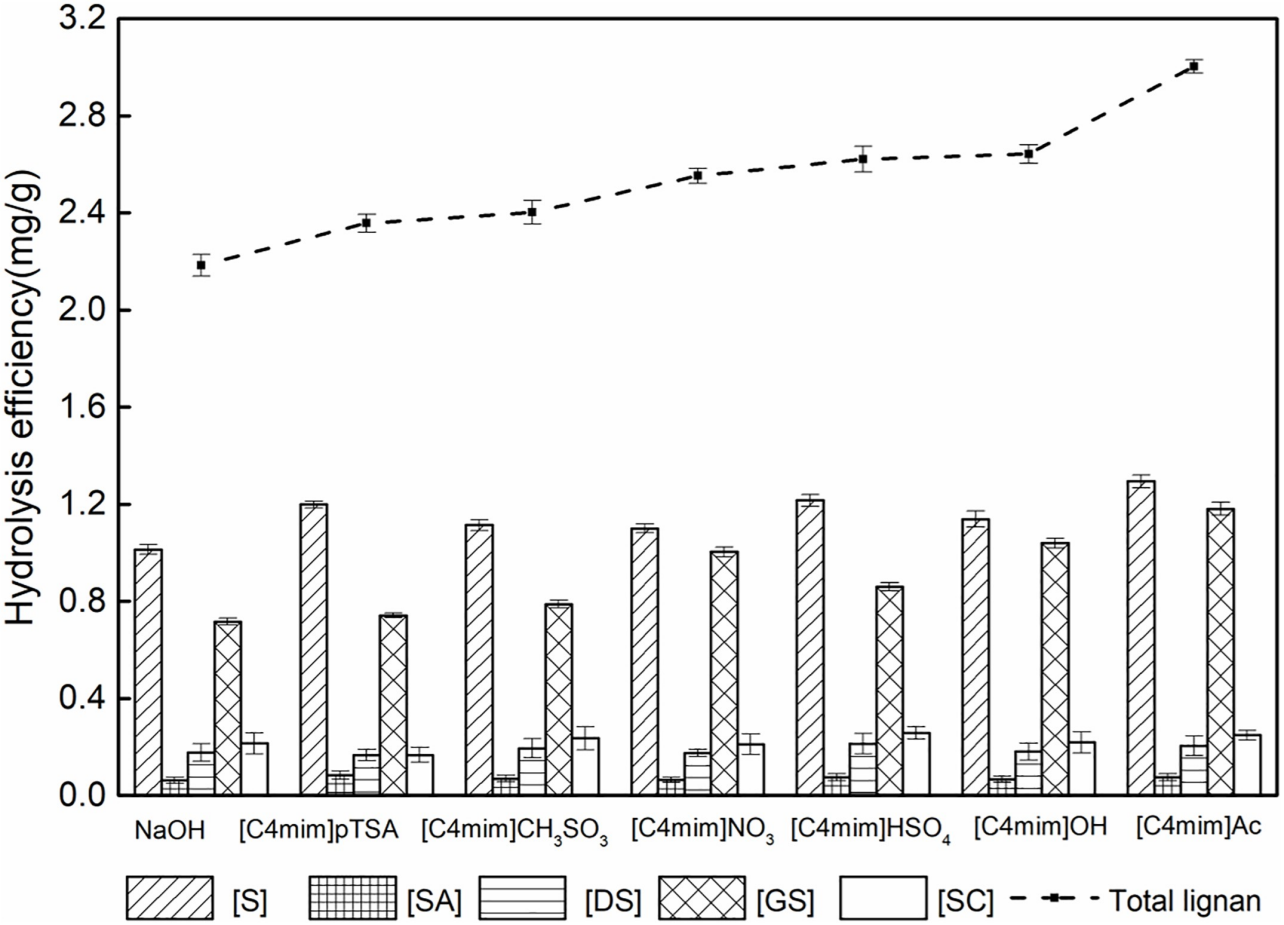
Hydrolysis efficiency of EBCLs with different catalysts.

Basic ILs can be divided into Lewis basic ILs and Brönsted basic ILs. Lewis defined bases according to their capability of donating an electron pair. Brönsted defined bases according to their ability to accept a proton. ILs containing basic anions can behave as base catalysts [4]. Therefore, the selected ILs [C4mim]OH (Brönsted base) and [C4mim]Ac (Lewis base) were examined, the inorganic base NaOH and acid ILs [C4mim]pTSA, [C4mim]CH_3_SO_3_, [C4mim]HSO_4_, [C4mim]NO_3_ was used as a reference, selection of the most favorable IL was based on the hydrolysis efficiency. The hydrolysis efficiencies of all the catalysts tested in this study are shown in Fig 3. In each test, *S*. *chinensis* extract was mixed with ILs or NaOH and then subjected to 600 W microwave irradiation for 20 min. Compared with NaOH, which achieved an average hydrolysis efficiency of FBCLs of only 2.18 mg g^−1^, [C4mim]Ac showed a substantial improvement of hydrolysis efficiency to 3.01 mg g^−1^. This increase of hydrolysis efficiency was achieved because the acidic hydrogens in the imidazolium ring of [C4mim]Ac (Fig 1), particularly C(2)-H, can form hydrogen bonds with cellulose, which suppresses the plant cell wall biomass recalcitrance effect and increases the accessibility of BCLs. The anion of [C4mim]Ac is an electron-pair donor and weak base (the acidity of ILs was shown in Table 1) that hydrolyzes to form nucleophilic hydroxide ions (OH^−^) continually [35], which hydrolyze EBCLs into FBCLs. Therefore, [C4mim]Ac was selected for the hydrolytic process of EBCLs because its hydrolysis efficiency was the highest of the investigated catalysts.

**Table 1:** Determination of acidity by Hammett acidity functions.

	[C4mim][NO_3_]	[C4mim][CH_3_SO_3_]	[C4mim][pTSA]	[C4mim][HSO_4_]	[C4mim]OH	[C4mim]Ac
I	0.72	0.78	0.86	0.62	0.89	0.92
IH^+^	0.28	0.22	0.14	0.38	0.11	0.08
*H*_*0*_	-4.11	-3.97	-3.74	-4.32	-3.62	-3.46
pH	5.68	4.87	4.06	2.48	9.73	7.74

**Fig 4:**
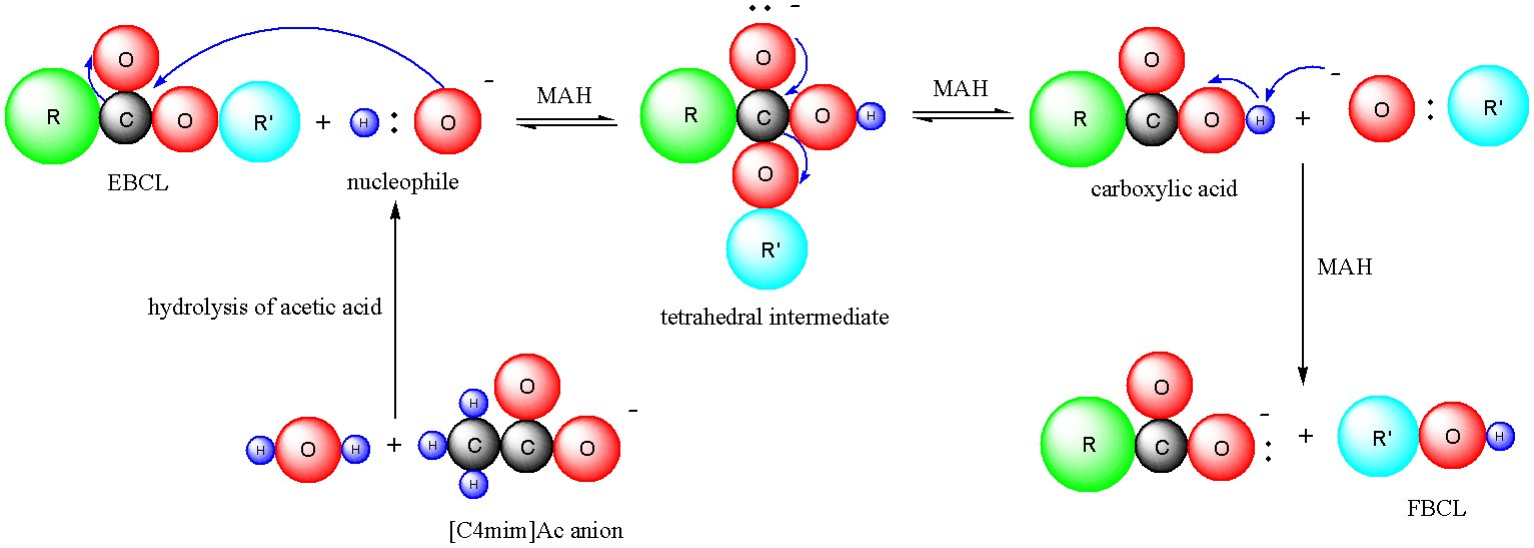
Mechanism of EBCL hydrolysis into FBCLs in [C4mim]Ac solution during MAH.

Fig 4 shows the proposed mechanism of EBCL hydrolysis to form FBCLs through the action of [C4mim]Ac during MAH. First, the acetate anion of [C4mim]Ac is hydrolyzed into the weak acid CH_3_COOH and OH^−^. The nucleophilic OH^−^ attacks the electrophilic carbon of the EBCL ester C = O, breaking the π bond and forming a tetrahedral intermediate. The intermediate collapses to reform the C = O group, resulting in the loss of the leaving group R’O^−^ and formation of a carboxylic acid (RCO_2_H). Finally, an acid/base reaction occurs where the RO^−^ functions as a base and deprotonates the RCO_2_H, which reaches equilibrium very quickly.

#### 3.2.2 Single factor analysis of the hydrolytic process with [C4mim]Ac

**Fig 5:**
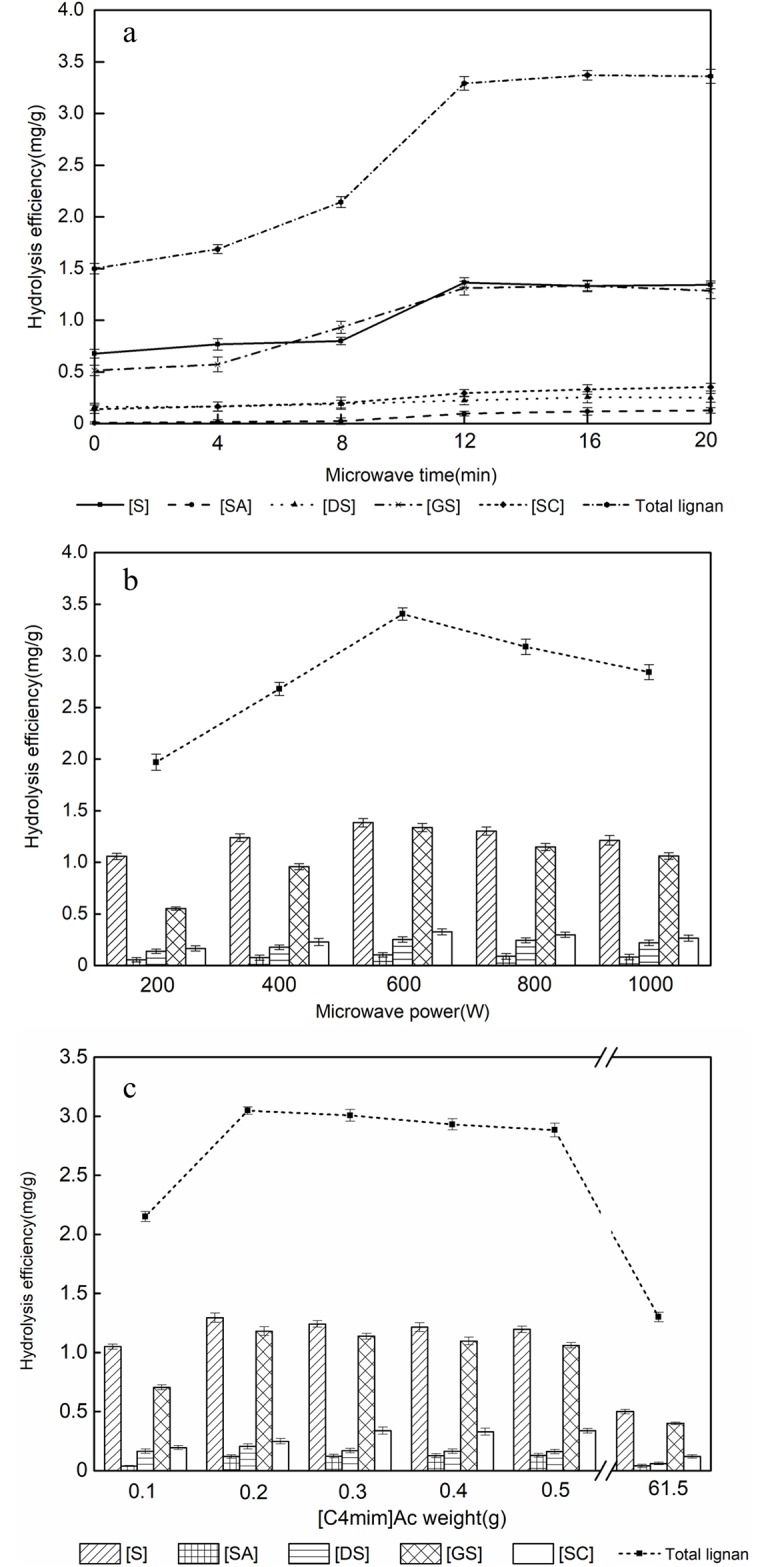
Effects of microwave time (a), microwave power (b), and [C4mim]Ac loading (c) on the hydrolysis efficiency of FBCLs.

*3*.*2*.*2*.*1 Effect of microwave heating time on hydrolysis efficiency*. Fig 5a depicts the hydrolysis efficiency of BCL extracts mixed with [C4mim]Ac and heated by 600 W microwave irradiation for different periods. As the microwave treatment time was lengthened from 0 to 12 min, the hydrolysis efficiency increased markedly. As the treatment time was extended from 12 to 20 min, the efficiency approached saturation. This indicated that microwave irradiation for a long period may result in carbonization of the raw materials because of internal overheating, the isomerization of FBCLs, and excess energy expenditure. Therefore, a microwave duration of 12 min was used in subsequent experiments.

*3*.*2*.*2*.*2 Effect of microwave power on hydrolysis efficiency*. To examine the effect of the microwave power on the hydrolysis efficiency, BCL extract was mixed with [C4mim]Ac and then hydrolyzed by microwave irradiation for 12 min at a microwave power of 200, 400, 600, 800, or 1000 W. Fig 5b reveals that the average hydrolysis efficiency increased with the microwave power up to 600 W. The heating curves with different power were showed in S1 Fig in the Supplementary Information. Above 600 W, the average hydrolysis efficiency of FBCLs decreased slightly. Increasing the microwave power results in a drastic increase of reaction temperature. The results indicated that high-power microwave irradiation (>600 W) may result in carbonization of raw materials through internal overheating and the isomerization or thermal instability of FBCs. In addition, the specific heat capacity increases with the reaction temperatures [36]. Thus, the microwave power of 600 W satisfied the conditions of high yield and low energy consumption. Therefore, an irradiation power of 600 W was selected for further experiments.

*3*.*2*.*2*.*3 Effect of catalyst loading on hydrolysis efficiency*. Catalyst loading is an important parameter in hydrolysis reactions; higher catalyst loading means that the system reaches equilibrium sooner because of the increased total number of basic sites available for the reaction [37]. The hydrolysis procedures were carried out in different solutions with different IL loadings to determine the optimal IL loading for MAH hydrolysis of the five kinds of EBCL. Fig 5c presents the hydrolysis yields for systems with different IL loading treated by MAH for 20 min. The optimal catalyst loading was 0.20 g per 50 mL of target solution. Less than 0.2 g may lead to incomplete hydrolysis and more than 0.2 g of catalyst resulted in unnecessary waste. When 0.20 g of [C4mim]Ac (X_IL_ = 0.00036) was mixed with 50 mL of *S*. *chinensis* extract, the IL molecules will not form ion clusters and the solution also has low viscosity and high diffusivity (refer to Section 3.1). In Fig 5c, we have added the data of FBCL extraction efficiency when the molar fraction of water was 0.9 in Fig 2 ([C4mim]Ac was 61.5 g). The results showed that extraction efficiency of FBCL with higher water contents is higher than that of the higher [C4mim]Ac content. The concentration of ionic liquids is not the higher, the better, and it is indirectly proved that the water molecular is contributed to the hydrogen bonds formation under the clusters concentration of Ionic liquid. We established a connection between the clusters concentration of Ionic liquid (Fig 2) and the extraction solvent concentration of Ionic liquid (Fig 5c).

### 3.3 Evaluation of different extraction procedures

#### 3.3.1 Surface morphology of *S*. *chinensis* fruit

**Fig 6:**
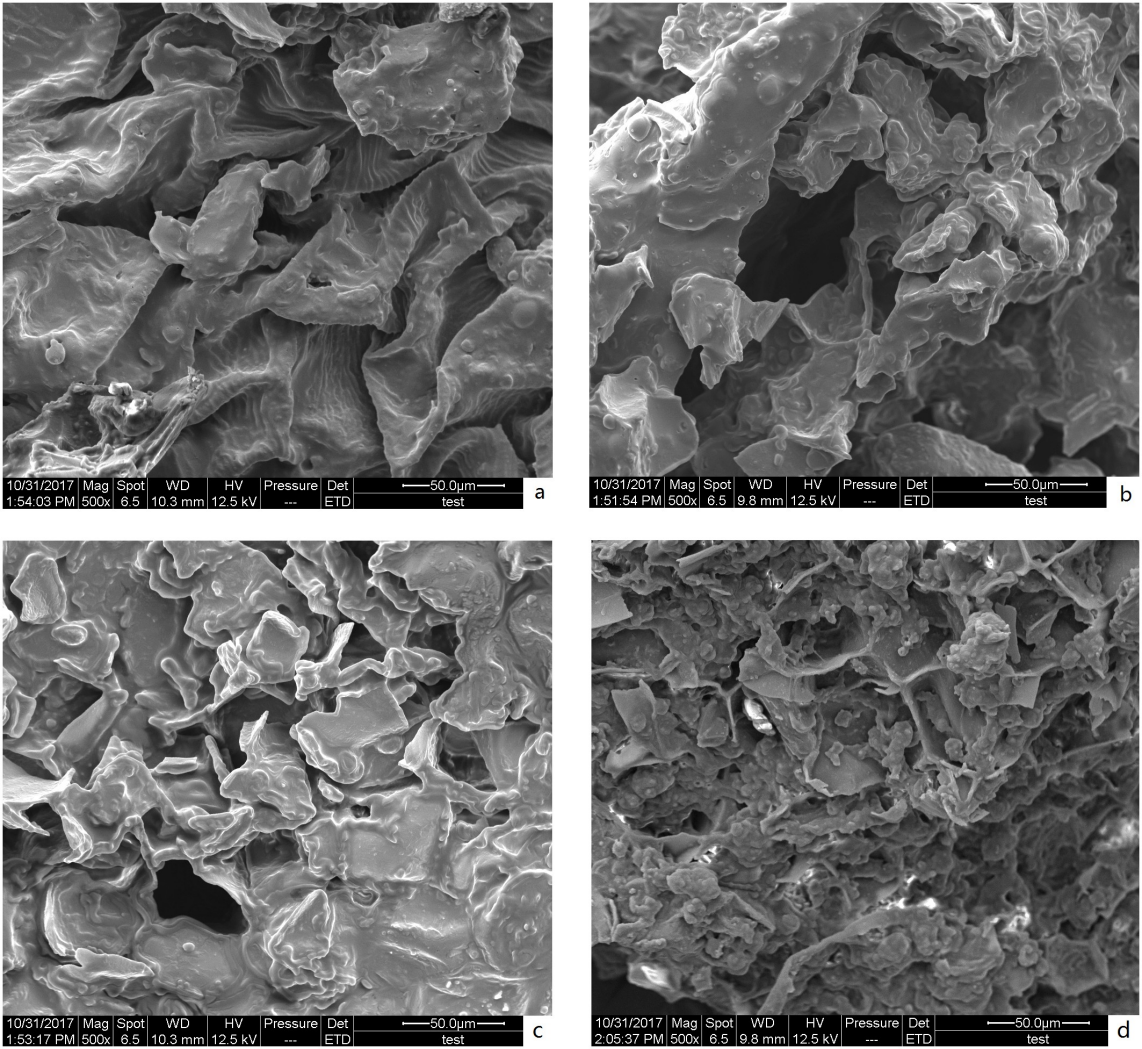
SEM images of *S*. *chinensis* fruit raw materials (a) and *S*. *chinensis* fruit residue after treatment by refluxing in IL (b), MAH in ethanol (c), and MAH in IL solution (d).

The different catalysts and extraction processes affected the surface morphology of *S*. *chinensis* fruit. SEM images of the fruit before and after the different extraction methods are provided in Fig 6. The images show that the untreated *S*. *chinensis* fruit (Fig 6a) has major cell structures that are relatively intact. Fig 6b and 6c, and d show SEM images of *S*. *chinensis* fruit after treatment by reflux in IL for 120 min, MAH in ethanol for 12 min, and IL-MAH exposure for 12 min, respectively. The cell structure was disrupted markedly after the extraction process. Most of the cells became atrophic, ruptured, and appeared wrinkled. After IL-MAH treatment (Fig 6d), the cell walls of *S*. *chinensis* fruit were highly damaged compared with those following treatment by refluxing in IL (Fig 6b) and MAH in ethanol (Fig 6c). Thus, it is conceivable that, as a combined result of [C4mim]Ac severely damaging plant cell walls and microwave irradiation continuously inducing internal thermal stress in the plant cells, the pressure build-up within the cells and membranes could exceed their capacities for expansion and cause their rupture, releasing EBCLs into the [C4mim]Ac solution, which facilitates their hydrolysis to FBCLs.

#### 3.3.2 Chemical changes in the cell walls of *S*. *chinensis* fruit after IL-MAH treatment

**Fig 7:**
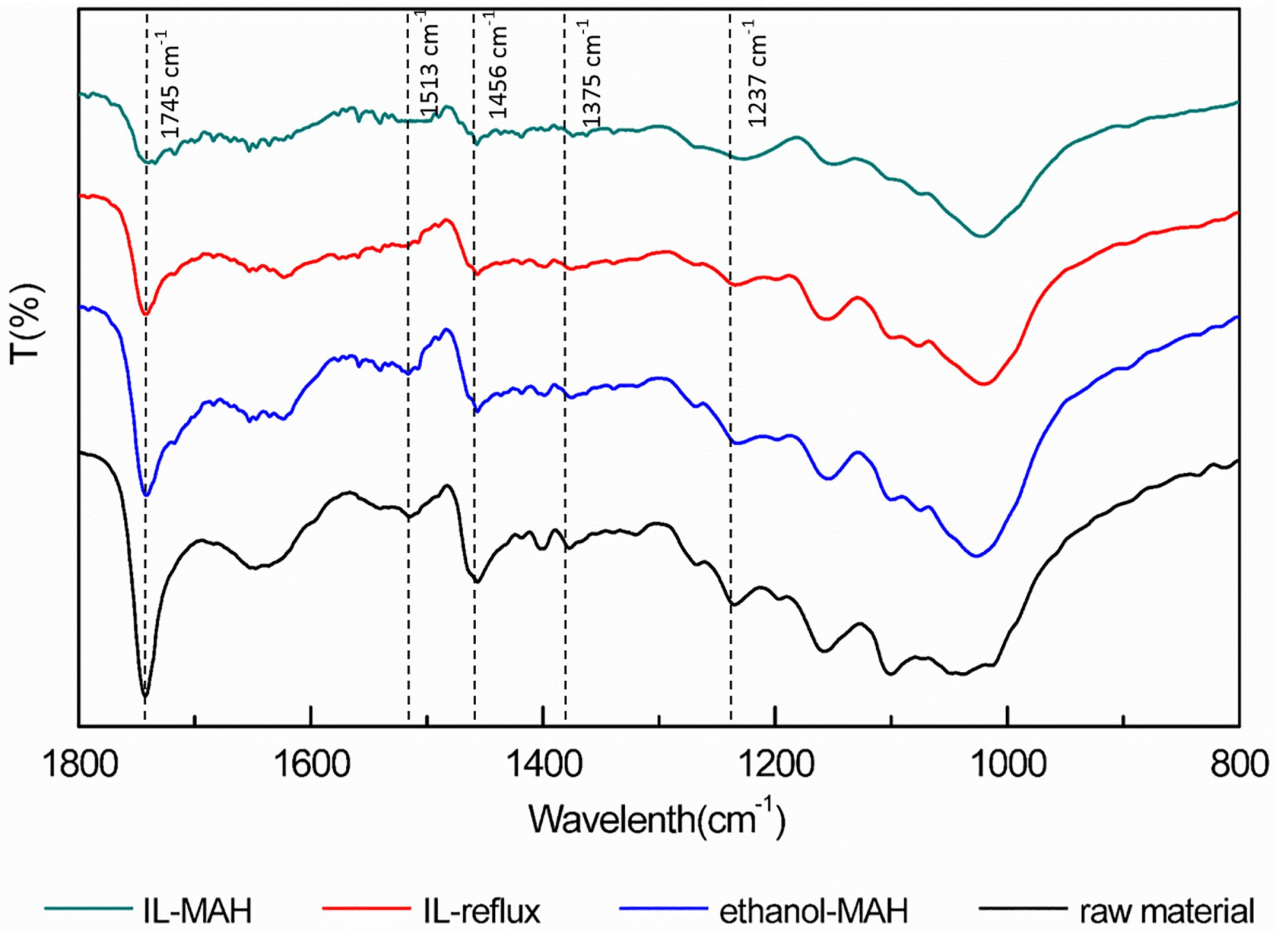
FTIR spectra of *S*. *chinensis* fruit residues before and after extraction by different extraction.

The solid residues of *S*. *chinensis* fruit before and after the different extraction procedures were qualitatively analyzed by FTIR spectroscopy (Fig 7). The region from 800 to 1400 cm^−1^ is associated with cellulose and hemicellulose structures. The peaks at 1237 and 1375 cm^−1^ attributed to acetyl C-O stretching and C-H deformation of the cellulose and hemicellulose structure were almost absent after the IL-MAH treatment. This implies that cellulose and hemicellulose were effectively deacetylated by IL-MAH treatment [38]. The peak at 1745 cm^−1^ originates from the complex linkages between hemicellulose and lignin, such as ester-linked acetyl, feruloyl, and p-coumaroyl groups [39]. For the IL-MAH treated cell particles, the intensity of the peak at 1745 cm^−1^ was much weaker than that before treatment, indicating that these linkages were broken during IL-MAH treatment. The absorption at 1456 cm^−1^ is attributed to C-H bending of lignin and that at 1513 cm^−1^ is related to lignin C = C aromatic stretching. These two peaks were much weaker after IL-MAH treatment compared with those of the raw *S*. *chinensis* fruit, providing clear evidence that lignin was efficiently removed by the IL-MAH process [40].

**Fig 8:**
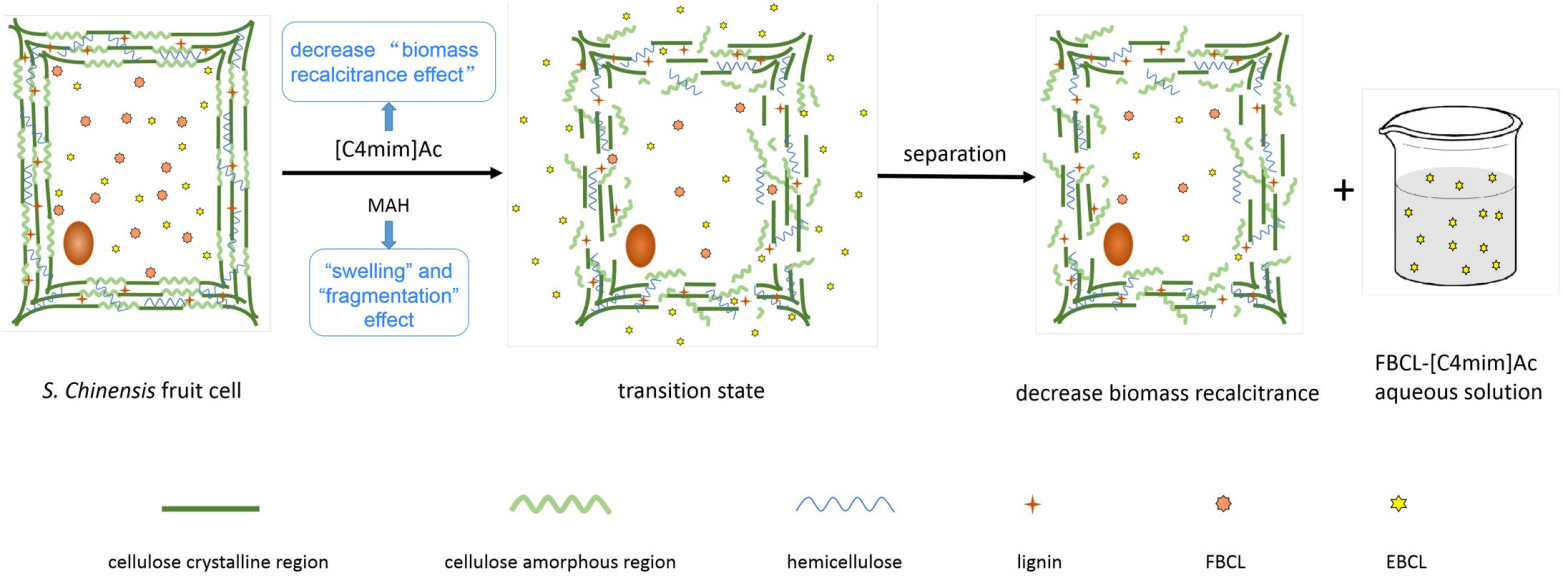
Contributions of an IL and MAH to suppressing the plant cell wall recalcitrance effect.

**Fig 9:**
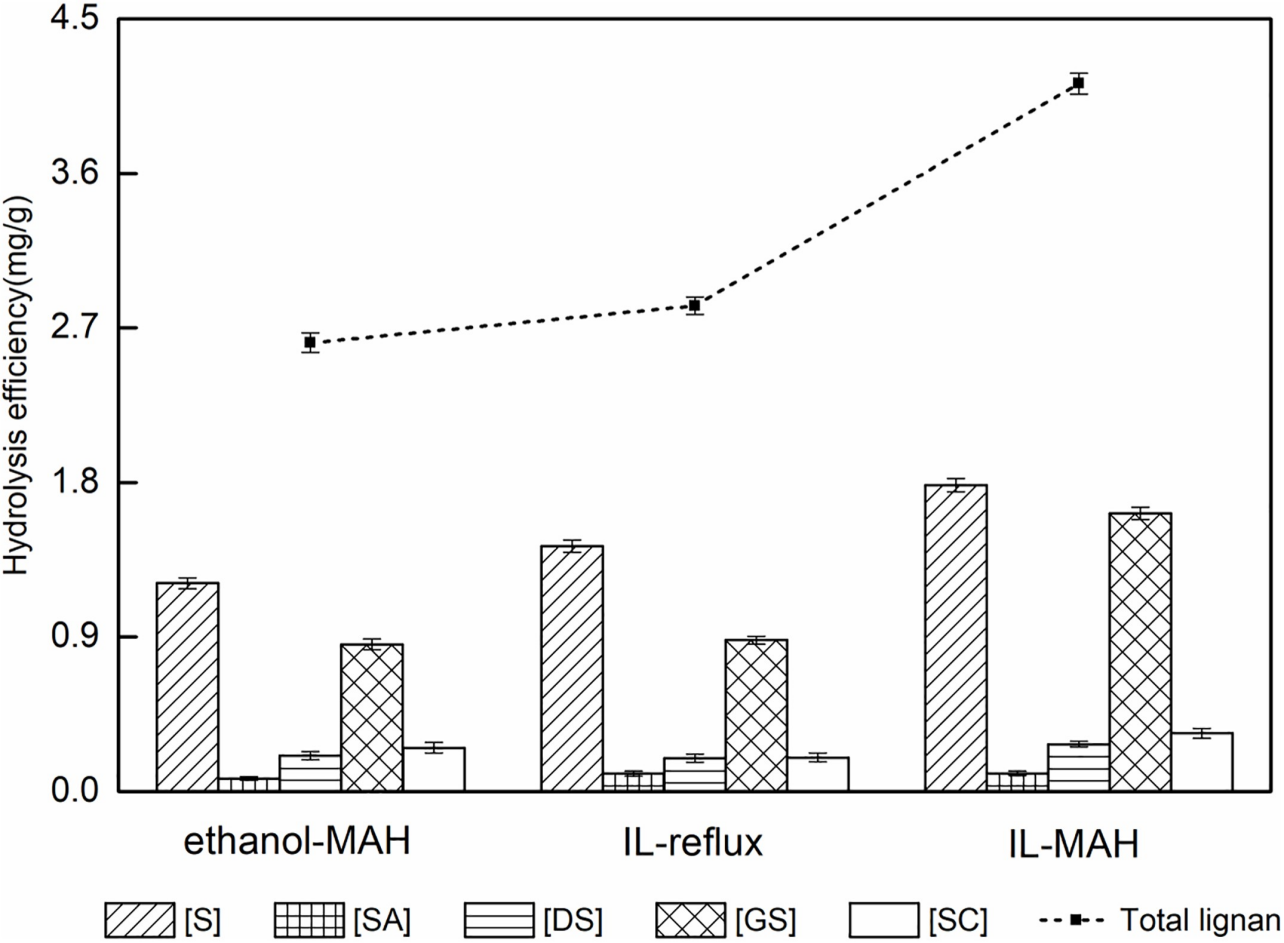
Yields of FBCL with different extraction methods.

Imidazolium ILs with carboxylic acid anions possess high basicity, low melting points, low viscosities, and high hydrogen-bond acceptor abilities. As a result, such ILs can facilitate the dissolution of plant matter and aid solution penetration into the biomass interior. Fig 8 depicts the roles of [C4mim]Ac and thermochemical pretreatments in the chemical and structural features of plant cell walls. [C4mim]Ac degrades the cross-linked matrix of lignin and hemicelluloses that embed the cellulose fibers, disrupts hydrogen bonds in crystalline cellulose, attacks the amorphous region of cellulose, and increase the porosity and surface area of cellulose to facilitate subsequent BCL extraction [41]. When MAH is applied to the lignocellulosic feedstock from *S*. *chinensis* fruit, selective heating of the more polar regions creates hot spots within the heterogeneous material [42]. When lignocellulosic biomass is exposed to MAH heating, swelling and fragmentation occur within the biomass, resulting in the disruption of the lignocellulosic structure. This consequently results in an increase of surface area, decreases in the degree of polymerization and crystallinity of cellulose, and the depolymerization of lignin, which ultimately improves the accessibility of BCLs [43]. Therefore, IL-MAH was the best method to extract FBCL from *S*. *chinensis* fruit which extraction efficiency up to 4.12±0.37 mg g^−1^ (Fig 9).

## 4. Conclusions

FTIR and Raman spectroscopic analyses indicated that ILs with a higher water content (>0.6) will not form ion clusters, so an IL with a low concentration of water, which has low viscosity and high diffusivity, was used to d crease biomass recalcitrance during extraction of BCL and as a catalyst for EBCL hydrolysis. The optimum parameters to treat a 5.0 g sample were an irradiation power of 600 W, microwave extraction time of 12 min, and 0.2 g of [C4mim]Ac. The yield of FBCLs under the optimum conditions was 4.12±0.37 mg·g^−1^. FTIR spectroscopic and SEM analyses of *S*. *chinensis* solid residues obtained after treatment by different methods indicated that alkaline IL-MAH was the most effective method to treat *S*. *chinensis* fruit. During the IL-MAH process, the lignocellulosic biomass of the *S*. *chinensis* fruit cell wall was exposed to microwave irradiation, which induced swelling and fragmentation within the lignocellulosic structure, resulting in the disruption of the cell wall. In addition, [C4mim]Ac also degraded the cross-linked matrix of lignin and hemicelluloses that embed the cellulose fibers, disrupted hydrogen bonds in crystalline cellulose, broke the ester linkage between lignin and xylan, and increased the porosity and surface area of cellulose to aid subsequent extraction and ester hydrolysis. A mechanism of EBCL hydrolysis in alkaline IL solution was also proposed. In the future, task-specific ILs with designable structures that can be selected according to the specific needs of a reaction, especially natural product structure modification, is anticipated to flourish.

## Supporting information

S1 Fig Heating curves with different microwave powers. (DOCX)

S1 Data (ZIP)
