# Supplementary material for: Decoding biomass recalcitrance: Dispersion of ionic liquid in aqueous solution and efficient extraction of lignans with microwave magnetic field
Source: PLoS One. 2020 Feb 21;15(2):e0226901. doi: 10.1371/journal.pone.0226901 (PMC7034798; doi:10.1371/journal.pone.0226901)
Supplement: S1 Fig — (DOCX) [file pone.0226901.s001.docx]

**Supplementary Information**





Figure S1 Heating curves with different microwave powers
